# Towards a Swiss health study with human biomonitoring: Learnings from the pilot phase about participation and design

**DOI:** 10.1371/journal.pone.0289181

**Published:** 2023-07-31

**Authors:** Réjane Morand Bourqui, Semira Gonseth Nusslé, Natalie von Goetz, Caroline Veys-Takeuchi, Claire Zuppinger, Yoanne Boulez, Nolwenn Bühler, Laurence Chapatte, Christine Currat, Aline Dousse, Vincent Faivre, Oscar H. Franco, Julien Virzi, Martine Bourqui-Pittet, Murielle Bochud

**Affiliations:** 1 Health Protection Directorate, Federal Office of Public Health, Bern, Switzerland; 2 General Direction, Center for Primary Care and Public Health, Unisanté, Lausanne, Switzerland; 3 Federal Institute of Technology (ETH Zurich), Zurich, Switzerland; 4 Institute of Social Sciences, University of Lausanne, Lausanne, Switzerland; 5 Swiss Biobanking Platform, Epalinges, Switzerland; 6 Institute of Social and Preventive Medicine, University of Bern, Bern, Switzerland; Iowa State University, UNITED STATES

## Abstract

**Background:**

A large-scale national cohort aiming at investigating the health status and determinants in the general population is essential for high-quality public health research and regulatory decision-making. We present the protocol and first results of the pilot phase to a Swiss national cohort aiming at establishing the study procedures, evaluating feasibility, and assessing participation and willingness to participate.

**Methods:**

The pilot phase 2020/21 included 3 components recruited via different channels: a population-based cross-sectional study targeting the adult population (20–69 years) of the Vaud and Bern cantons via personal invitation, a sub-study on selenium in a convenience sample of vegans and vegetarians via non-personal invitation in vegan/vegetarian networks, and a self-selected sample via news promotion (restricted protocol). Along with a participatory approach and participation, we tested the study procedures including online questionnaires, onsite health examination, food intake, physical activity assessments and biosample collection following high-quality standards.

**Results:**

The population-based study and the selenium sub-study had 638 (participation rate: 14%) and 109 participants, respectively, both with an over-representation of women. Of altogether 1349 recruited participants over 90% expressed interest in participating to a national health study, over 75% to contribute to medicine progress and help improving others’ health, whereas about one third expressed concerns over data protection and data misuse.

**Conclusions:**

Publicly accessible high-quality public health data and human biomonitoring samples were collected. There is high interest of the general population in taking part in a national cohort on health. Challenges reside in achieving a higher participation rate and external validity. For project management clear governance is key.

## Introduction

Health data are not only essential for research purposes, but also for evidence-based public health policy–they form the basis for public health decisions and interventions. In Switzerland, health data on the general population are mostly fragmented, local and heterogeneous. Already in 2011, the OECD pointed out in its review of the Swiss health system a poor health information system, needing appropriate effort into collecting the information necessary to generate evidence [1]. Switzerland’s demographics and life expectancy–one of the highest in the World [2]–are such that some scientists predict a grey tsunami [3]: the proportion of the elderly in the population will drastically increase in the coming decades from 18% currently [4] to up to 30% in 2050 [5]. Although Switzerland’s global health status is fairly good compared to the neighboring countries [6], socioeconomic inequalities in health are substantial [7, 8]. Another future challenge lies in the health impacts of climate change. For Switzerland, as a middle-European country, experts announce for instance a rise in communicable diseases, and in the lethality and morbidity of heat stress and weather-related disasters, together with a worsening of health inequalities consecutive to migrations and economic crisis [9–11].

These challenges ahead underline the need for good quality health data for both public health monitoring and research. We need studies that can take into account the broad and interconnected cumulative health effects of life-course exposures, be they environmental, related to lifestyle, due to toxic compounds, or “social exposures” such as social inequities [12]. The exposome–a concept encompassing all exposures on an individual, from conception and onward–can be studied at different scales, from external general exposures down to specific molecular alterations. Population-based cohort studies investigating the exposome constitute a method of choice for epidemiology and public health: they enable a better understanding of the determinants of health and disease and a monitoring of the impact of public health policies. Exposome-oriented research has the potential to make a breakthrough in overcoming the limitations of conventional epidemiology, such as misclassification of exposures, complex interactions between exposures, and causal inference [13]. Remarkable examples of such ongoing studies are CONSTANCES in France [14], the UK Biobank [15, 16] and the LifeGene study in Sweden [17].

There is currently no large-scale national population-based cohort in Switzerland that would allow (1) identifying and monitoring relevant complex exposures and health needs of the Swiss population, (2) conducting targeted health research for preparing national health policies and assessing their effectiveness, as well as enabling the development of selected indicators (e.g. reference values). The data available within existing cohorts was collected with different purposes and protocols, which results in a lack of interoperability. Furthermore, their sample size limits the ability to explore the impact of chronic exposure to stressors such as toxic substances, disease vectors, or noise. The absence of dedicated public funding for such long-term large-scale national projects results in a lack of sustainable nationwide infrastructures. In order to fill this gap, a large-scale population-based national cohort that would involve a large number of citizens—up to 100,000—is envisaged. Its aims are to preserve and improve the health of the Swiss population by providing high-quality longitudinal data allowing health and exposure monitoring and advances in research, which will constitute the basis of evidence towards strong health policies. Here we present the study protocol and participation results of a pilot phase, initiated by the Federal Office of Public Health (FOPH), in collaboration with academic public health institutions in Switzerland, along with attitudes towards participation in a national cohort. The aims of the pilot study are to test selected feasibility aspects of a national cohort, in terms of participation, infrastructure, centralized biobanking of collected biosamples and coordination, as well as to provide scientific evidence on specific environmental exposures of concern in Switzerland.

## Study design and methods

The current project includes 3 main interrelated components: (1) a cross-sectional, population-based, observational study constructed as a pilot phase for a large-scale national population-based cohort including human biomonitoring (HBM), (2) a sub-study focusing on a selected nutritional aspect of relevance for public health surveillance (Selenium sub-study) and (3) a study on a self-selected sample undergoing a restricted protocol.

The project has four main objectives: (1) to investigate the technical feasibility of a Swiss population-based cohort and establish selected components of the study infrastructure, as well as standard operating procedures in two centers from two different linguistic regions; (2) to assess the willingness of the Swiss population to participate in such a study and associated obstacles; (3) to evaluate the prevalence of targeted environmental exposures of concern by analysing biosamples (glyphosate, metals, and per- and polyfluoroalkyl substances) and their associations with relevant health outcomes; (4) to test the feasibility of adding sub-studies to the main population-based cohort.

The study infrastructure included common IT systems for data and sample collection, a central biobank for sample storage and a quality management system. During the pilot study, the governance was also tested, along with the interplay between different stakeholders (government, research, participants).

### Study participants

**Fig 1:**
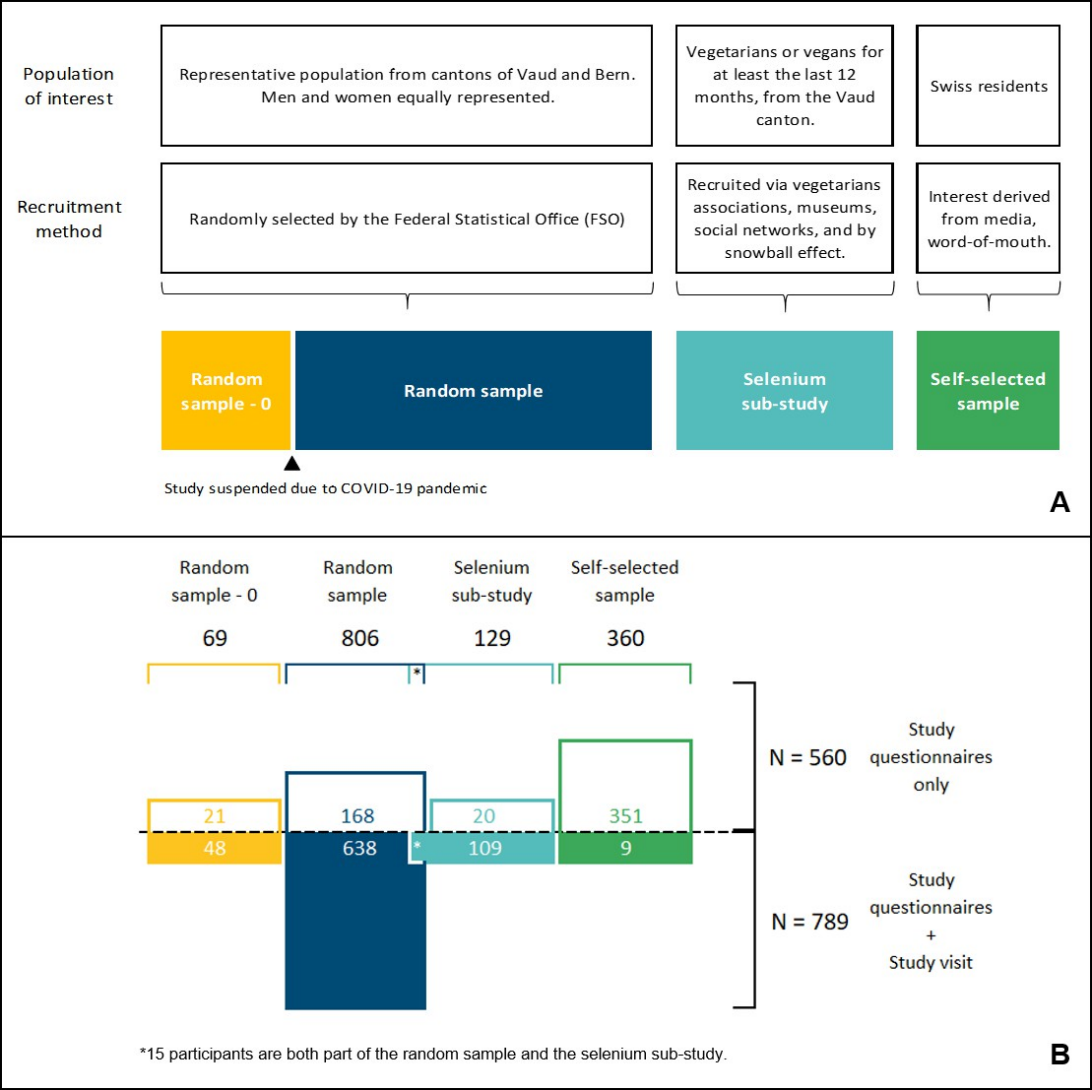
Components of the project, including target populations and recruitment methods. Panel A: Study samples and their respective population of interest and recruitment method; Panel B: Number of participants per level of participation and recruitment channel.

Fig 1, panel A, describes the 3 main components of the project, their target population and recruitment procedures. Before completing the online questionnaires, all participants filled in an informed e-consent. Participants invited to the study visit filled in an additional written informed consent at the study center (Fig 1).

#### Random samples

We distinguish the “random sample -0”, drawn end of 2019, with a recruitment initiated in January 2020 and suspended in March 2020 due to the COVID-19 pandemic, and the “random sample”, drawn for the study restart in October 2020. Both samples were randomly drawn from the sampling frame of the Federal Statistical Office (FSO) according to the Ordinance on Statistical Surveys, Art. 13c, al. 2 (SR 431.012.1), but in different drawings. Both random population drawings were representative of the population aged 20 to 69 years of the cantons of interest.

Because of the pandemic, recruitment was disrupted for participants from the “random sample -0”. These persons received no reminder letter, no recruitment calls and many of them could not be received at the study visit. Therefore, the “random sample -0” was not used for calculating the participation rate, and received a separate denomination, but the samples and data were kept in case that they would be useful for any follow-up studies.

For the random sample for the study restart in October 2020 , a total of 4’542 persons with valid addresses were reached via postal mail (2’226 in canton Vaud, 2’316 in canton Berne), with a recruitment target of up to 500 study participants per canton. Eligibility criteria were age between 20 and 69 years, primary residence in the canton, ability to answer questionnaires in German, French or Italian, and capacity of discernment. Exclusion criteria were institutionalized individuals, and refusal to be informed in case of relevant findings that may have had a health impact.

Following invitations by postal mail, two reminder letters were sent in case of non-response after 3 to 4 weeks. In each canton, the mailing was subdivided into five mailing waves of about 500 addresses over the study period, in order to reflect a full year cycle in the study visits.

In the invitation mailing, the prospects were asked to log onto the study website (https://www.etude-sur-la-sante.ch) with a unique identifier code to access the online study questionnaires., either in French, German or Italian. Alternatively, the invited participants could request the paper version of the questionnaires. Once the questionnaires were completed, the research team would call the participant to schedule a visit at the study center (phone number provided via the personal data questionnaire). Whenever the phone number was available from the Statistical Office data (for about 2 out of 5 prospects), prospects who had not responded to the invitation were called to encourage participation, either by the research team (Berne) or by a trained call center (Vaud). When possible, prospects expressing their unwillingness to participate were administered a brief non-respondent questionnaire over the phone on their sociodemographic data, selected health outcomes and risk factors.

#### Selenium sub-study

A sub-study on selenium (Se) targeted persons following a vegetarian or vegan diet. Given the expected low number of vegans and vegetarians in the random sample, we used other recruitment channels such as specialized businesses, vegetarians’ associations, museums, social networks, as well as snowball effect within the investigators’ and participants’ networks. Individuals following a vegan or vegetarian diet for at least 12 months (pesco- or ovo-lacto- vegetarians admitted), aged between 20 and 69 and resident in the Vaud canton were included. They underwent the exact same procedures as the randomly selected participants with the addition of a modified food-frequency questionnaire [18–20] and an extra blood sample for selenium and B12 vitamin measurements. The selenium sub-study sample included 109 participants.

#### Self-selected sample

In addition, all Swiss residents aged 20 to 69 years were eligible to spontaneously fill in the study questionnaires on the study website. This option was communicated through media and directly on the study website. The participants from the self-selected sample were in principle not invited to the study visit.

### Data sources and measurements

The topics covered in the self-administered questionnaires are listed in (Table 1).

**Table 1:** General topics covered by the study questionnaires and interviews.

			Random sample—0	Random sample	Selenium sub-study	Self-selected sample
Name	Format	Subject				
**Study questionnaires**	Self-administered	Opinion towards health research	✔	✔	✔	✔
		Environment & Lifestyle	✔	✔	✔	✔
		Medical History	✔	✔	✔	✔
		Quality of Life	✔	✔	✔	✔
		Coronavirus		✔	✔	✔
**Study visit**	Interview questions	Environment & Lifestyle	✔	✔	✔	
		Preparedness to contribute to a health study	✔	✔	✔	
	Performed by study nurses	Health examination	✔	✔	✔	
	Performed by study nurses	Biosample collection	✔	✔	✔	
**Post-visit assessment**	Self-administered questionnaire	Food Frequency questionnaire			✔	
	Wearable device	Accelerometry	✔	✔	✔	
	Smartphone App	Food consumption (MyFoodRepo)		✔	✔	
**Non-respondent questionnaire**	Self-determined by phone for prospective participants who refused to take part in the study	Brief socio-economic data and attitude towards research	✔	✔		

After having completed the online questionnaires, the participants came to a study center for a 2.5 hour visit, which included the following steps: interview on specific lifestyle and occupational exposures; anthropometric measurements and body composition assessment by bioimpedence (Tanita^®^ MC-780MA-N II); blood pressure measurements by sphygmomanometer (Omron^®^ M3); spirometry (Easyone Air^®^); handgrip strength (Jamar^®^ dynamometer); and a sample of expired air for oxidative potential measurements [21] (in Vaud only). Optionally, accelerometers (Actigraph^™^ wGT3X-BT) were worn by participants volunteering to record their physical activity, and likewise optionally food intake was self-recorded by the participants using a barcode- and picture-led phone application (MyFoodRepo App [22]), both during 8 consecutive days following the study visit (Table 2).

**Table 2:** Description of the data collection procedures.

Tested systems	Details of procedures
Cardiovascular	Systolic and diastolic blood pressure (3 measurements), heart rate
Anthropometrics	Weight, height, and waist, hip, and neck circumferences
Bioimpedance analysis	Body composition: percentages of body fat, fat mass, fat free mass, muscle mass, total water mass, visceral fat level, bone mineral mass, physical constitution, extracellular and intracellular fluids, balance of the muscular mass (by segment), and basal metabolism
Frailty	Handgrip strength (3 measurements with the dominant hand)
Respiratory	Spirometry to assess lung function (including room temperature, room humidity and atmospheric pressure): forced vital capacity, peak expiratory volume in seconds, forced expiratory flow, peak expiratory flow, forced expiratory time, forced inspiratory vital capacity, peak inspiratory flow Sampling of exhaled air for Oxidative Potential in Exhaled Air measurement
Physical activity	Waist-worn accelerometer during 8 consecutive days: vertical and antero-posterior activity counts per minute, as well as vector magnitudes of the two axes
Diet	Food and beverage intake during 8 consecutive days self-recorded by the participant with a smartphone application

Capillary and venous blood, as well as spot urine were collected on site. Point-of-care analyses (Afinion 2, Abbott^®^) encompassed glycated hemoglobin, blood lipid profile, and urinary albumin/creatinine ratio. A simple blood formula, blood electrolytes, thyroid and renal functions, as well as anti-SARS-CoV-2 immunoglobulins G and ferritin were directly analyzed in the respective hospital laboratories (Table 3).

**Table 3:** Biobanked biospecimen (including pre-analytical steps) and laboratory analyses.

Biobanked samples		
Type of tube	Preparation step	Aliquot n°&type Central storage
Lithium-heparin for Trace metal Analysis 7.5 mL	No centrifugation	4x 0.7 mL whole blood
Blood Plasma EDTA-K 7.5 mL	Centrifugation 3000g/ 7min/ 20^°^C with brake	4x 0.7 mL plasma EDTA
		1x 0.7 mL buffy coat
Plasma Li-heparin 7.5 mL		4x 0.7 mL plasma Li-hep
Serum no gel tube 7.5 mL		5x 0.7 mL serum
		1x 2 mL microtube
Urine (no additive) 8.5 mL		8x 0.7 mL urine
**Total aliquots**		**27x aliquots**
**Direct laboratory analyses**		
Clinical chemistry in blood	Sodium, potassium, chloride, phosphate, total calcium and corrected-calcium, creatinine, cystatin C, total cholesterol, HDL- and LDL-cholesterol, triglycerides, albumin, ferritin, B12 vitamin *(nested sub-study only)*, thyroid-stimulating hormone, free T3 and free T4, anti-SARS-CoV-2 spike and nucleocapside IgGs	
Hematology	Total leucocytes, erythrocytes, platelets, hemoglobin, hematocrit, mean corpuscular volume, mean corpuscular hemoglobin, mean corpuscular hemoglobin concentration, red cells distribution width, average platelet volume, platelet distribution index, erythroblasts	
Spot urine	Sodium, potassium, chloride, phosphate, total calcium and corrected-calcium, urate, creatinine	
Point-of-care analyses in capillary blood and spot urine	Blood: glycated hemoglobin, total cholesterol, HDL- and LDL-cholesterol, triglycerides	
	Urine: albumin/creatinine ratio	
**Selected exposures**		
Metallomics	Multi-Metal panel analysis in whole blood: Al, Ag, As, Be, Bi, Cd, Co, Cr, Cu, Hg, I, Li, Mn, Mo, Ni, Pb, Pd, Pt, Sb, Se, Sn, Ti, U, V, Zn	
Pollutants	Per- and polyfluoroalkylated compounds in serum and glyphosate in spot urine	

### Pilot-specific outcomes

Feasibility in terms of participation was assessed by analyzing participation rates throughout the sending waves within the random population sample, and by examining the completion rate and the quality of the collected data.

In addition, a medical anthropologist conducted a qualitative study among a subsample of participants. For this study, several focus groups were organized with study participants who completed the study questionnaires (with or without attending a study visit) and 12 semi-directed individual interviews were conducted. Explored topics included (1) aspects related to the study design, (2) ethically and socially sensitive topics, such as the return of results, general consent or data protection, (3) expectations and concerns related to study, biomedical research, public health and environmental health. The focus groups results are presented in a dedicated manuscript [23].

## Study organization

### Centers

The study was carried out in two centers in parallel, in a French-speaking region (City of Lausanne, Canton of Vaud, VD) and a mainly Swiss-German speaking region of Switzerland (City of Berne, Canton of Berne, BE). In Vaud, the local research coordination team was located at the Center for Primary Care and Public Health, Unisanté, and study visits were conducted by the Clinical Research Center (CRC) of the Lausanne University Hospital (CHUV). In Berne, the local research coordination team was located at the Institute for Social and Preventive Medicine (ISPM), and visits were conducted by the Clinical Trials Unit (CTU) of the Berne University Hospital (Inselspital). Recruitment started in Vaud in January 2020, and was suspended in April 2020 due to the COVID-19 situation. Recruitment restarted in Vaud in October 2020 and in Berne in November 2020, and lasted until December 2021 in both cantons.

### Data and samples management

All questionnaires and the participant management tool were built on REDCap [24]. Metadata related to biospecimen collection and processing were recorded and securely stored within a laboratory management information system (SLIMS, Agilent^™^) managed by the Swiss Biobanking Platform (SBP). To ensure data protection, participants’ personal identifying data was collected and stored separately from research data.

Collected biospecimen were directly preprocessed into blood and urine aliquots. A temporary storage was made in the regional centers and then long-term storage was centralized in the Liquid Biobank Berne LBB [25]. In accordance with the informed consent, the data and samples are available for further analysis via the Unisanté website [26] and the NeXT catalogue from SBP [27]. The study is also listed in the Maelstrom catalogue [27].

### Quality management

To ensure comparability and quality of both data and biosamples, a quality assurance system was implemented during the pilot phase. This work was supported by the SBP as the national reference platform for biobanking activities. The standard operating procedures were harmonized with common work instructions for the study centers regarding sample collection, processing, transport and storage. The quality system also included a standardization of the staff training; a scheme of internal and external communication around the pilot phase, i.e. regular team meetings and regular information updates on the study website and in trimestral newsletters; and a detailed QMS documentation based on the study manual and derived working instructions.

SBP conducted an independent review of each site’s biobanking processes via its online Biobank SQAN tool [28]. This tool incorporates the requirements and recommendations of current standards (e.g. ISO 20387) and includes questions focused on governance, resource management and operational processes from sample collection to distribution. This review was supplemented by an analysis of key documents and a site visit. Some data, including dates and times of sample collection, processing, freezing, and storage, were analyzed to assess the consistency of data entries between the different systems. The time between collection and freezing was evaluated as an indicator of biosample quality.

### Funding, governance and management

The Swiss Federal Office of Public Health (FOPH) initiated the pilot study and gave an operationalization mandate to SBP, who has in turn commissioned the regional centers, i.e. the Center for Primary Care and Public Health located in the city of Lausanne in the canton of Vaud, and the Institute of Social and Preventive Medicine located in the city of Bern in the canton of Bern, respectively. In addition, the Swiss Tropical and Public Health Institute, located in the canton of Basel, was implicated in the elaboration of the study design, questionnaires, procedures and protocol. Funding primarily came from the Swiss government through its agencies (FOPH, Federal Office of Food Security and Veterinary Affairs (FSVO), Federal Office for the Environment (FOEN)), and from the involved institutions. Because of the pilot nature of the study, the institutions were involved in both the management (governing board) and the operational level (operational team) to integrate the respective interests of the partners. Both the governing board and the operational team included people from FOPH and the involved institutions.

### Nested sub-studies

Sub-studies were conducted with the aim of testing how a national cohort can become a research infrastructure able to respond to a wide range of public health and targeted research needs in a modular, flexible and efficient way.

A sub-study nested in the main study aimed at measuring the levels of blood selenium (Se) and B12 vitamin in participants following vegan or vegetarian diet using a convenience sample as described above. Selenium is an essential micronutrient that comes mainly from animal-based foods, suggesting that vegans and vegetarians are at higher risk of deficiency, which has multiple detrimental effects on health. This sub-study was a mandate of, and financed by, the FSVO.

Another sub-study was the integration of a novel sampling method for the measurement of oxidative substances in exhaled air (OPEA) [21] for a subset of participants, conducted by a research team from one of the involved partner institutions. This method allows determining the impact of selected pollutants or lung inflammation on various markers found in exhaled air.

### Statistical analyses

Data were collected between Feb 3^rd^, 2020 and December 15^th^, 2021 and analyzed with the statistical software R [29]. We present descriptive statistics, using counts and percentages together with bar graphs and radar plots to illustrate willingness or reluctance to participate in the different study components and across age groups. We used the Armitage trend test to explore the associations of selected answers to questionnaires across age categories.

### Ethics statement

Ethics approval: The protocol, questionnaires and procedures of this multicentric study was first approved on December 3, 2019, by the Ethics Committee of the research on human of the Canton of Vaud, Switzerland (number: 2019–01898).

Consent to participate: a written informed consent was obtained from each participant. An electronic informed consent was obtained for participants taking part to online questionnaires only.

Consent for publication: the participants’ consents cover the present publication.

## Results

### Study governance

The governance of the project was set up in order to allow regular interactions between all involved stakeholders with lean decision-making processes. The discussions between governmental agencies and researchers from academic institutions revealed different perspectives and focus areas, which highlights the need of clearly defined organization, roles and responsibilities of all involved stakeholders for a future large scale national cohort. An additional challenge was that the recruitment had to be stopped because of the COVID-19 pandemic, which led to a substantial delay in the project.

### Number of participants in each study component

A total of 1349 people completed the online questionnaires, including 69 persons from the random sample -0, 806 from the random sample, 129 from the selenium sub-study and 360 from the self-selected sample (Fig 1, panel B). A total of 789 participants completed the onsite study visit, including 48 from the random sample -0, 638 from the random sample, 109 from the selenium sub-study and 9 from the self-selected sample (Fig 1, panel B). Note that 15 participants are included in both the random sample and the selenium sub-study.

### Participation rate in the random population sample

**Fig 2:**
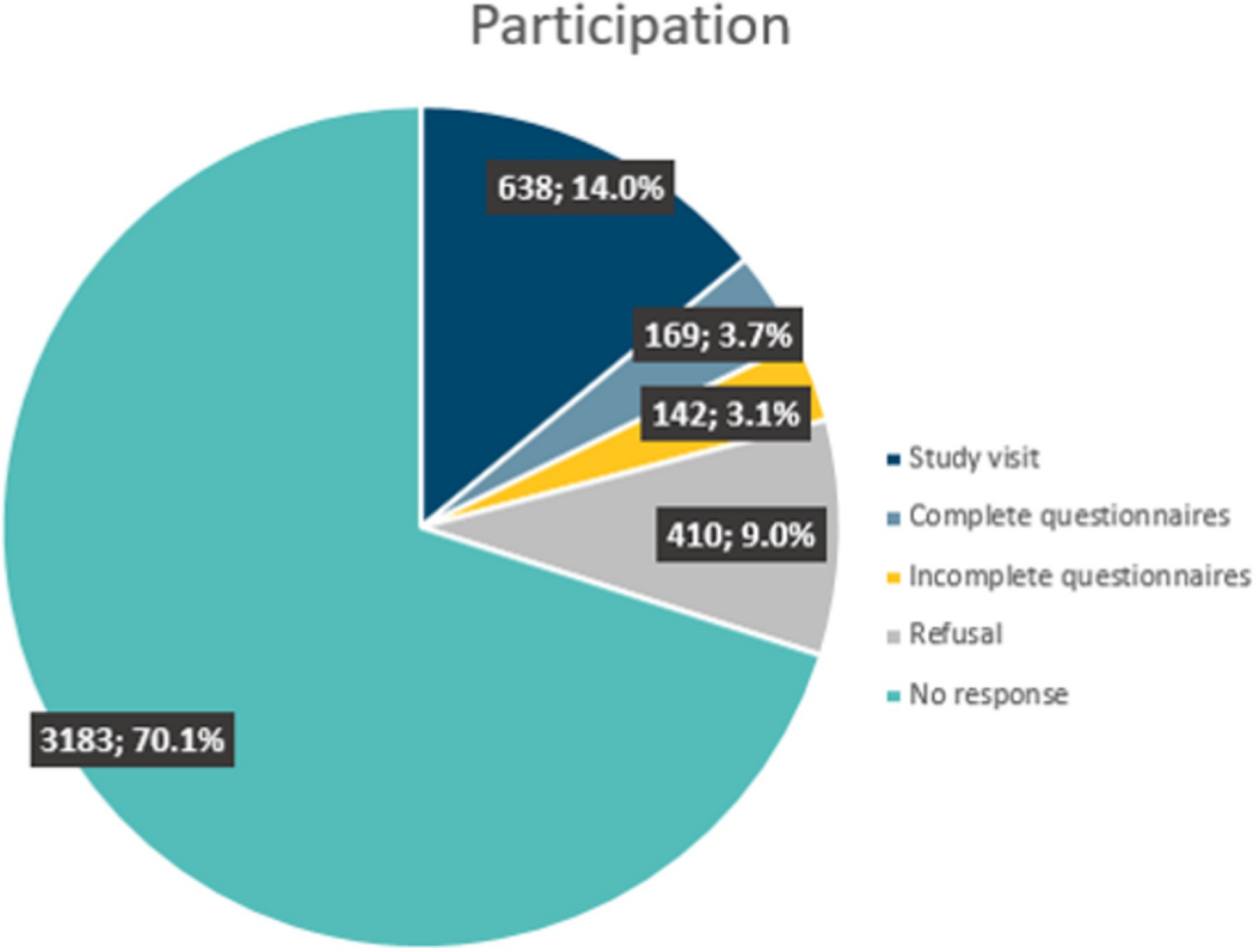
Participation rate to the population-based component (random sample). Pie chart of the participation based on a random population-based sample.

From the pool of randomly selected addresses initially received from FSO (N = 4’760), 4’542 addresses could be reached. Around 30% of invited persons responded to the invitation, with 9% refusal (N = 410) and a little more than 20% of the participants (N = 949) starting to fill in the questionnaires. Some left the questionnaires unfinished (N = 142) and some did not attend the study visit (N = 169), so that in the random sample, the final participation rate was 14.0% (number of persons attending the study visit [N = 638]/number of persons reached in the random sample [N = 4542]) (Fig 2).

We observed a gradual decline in the participation rates across the 5 mailing waves, with the highest rate of 17.2% for the 1^st^ wave (S1 Table) and the lowest (10.6%) for the 5^th^ wave. This is partly due to the fact that the intensity of phone calls was higher for the first mailing wave. We also observed differences in the participation rate depending on whether the reminders were based on letters or phone calls, with higher participation rates when phone numbers were available (17.2% vs 11.8%, p<0.01) (S2 Table). The number of phone call attempts was limited to 10 per prospect, since more phone calls did not improve the participation rate but rather annoyed the study prospects.

### Consent rates and completion rates of online questionnaires

For the Selenium sub-study and the self-selected sample the initial sample is unknown. Therefore, a participation rate cannot be calculated. In order to compare the willingness of participants recruited via the different channels, we compare the drop-outs at different stages of the study by departing from the baseline sample that was reached (random sample) or logged in (Selenium sub-study and self-selected sample). The e-consent completion rate was 20.9% (949/4’542) in the random sample, 59.7% (132/221) in the selenium sub-study and 50.7% (653/1’287) in the self-selected sample (Table 4). As most of the questions in the questionnaires were mandatory, the percentage of missing values in the questionnaires was low. However, we observed some drop-outs throughout the 5 study questionnaires, most probably due to the length of the questionnaires. In the random sample, out of 949 participants who completed the e-consent, only 807 (85%) completed all the questionnaires (Table 4). For the selenium sub-study and self-selected samples, the corresponding questionnaires completion rates were 98% (129/132) and 55% (359/653), respectively.

**Table 4:** Comparison of completion rates at different stages for the three recruitment channels.

Study stages	Random sample	Selenium sub-study	Self-selected sample
Baseline sample	4’542	221	1’287
(reached / logged-in)			
Complete e-consent	949 (20.9%)	132 (59.7%)	653 (50.7%)
Complete questionnaires	807 (17.8%)	129 (58.4%)	359 (27.9%)
Study visit	638 (14.0%)	109 (49.3%)	

Number and percentage of participants completing different stages of the study in the random, selenium sub-study and self-selected samples, after online registration via the study website (participants from the random sample had the option to send a postcard to register for paper questionnaires). The study visit was only proposed (by phone call) to the participants from the random or the selenium sub-study sample, once they had filled at least four questionnaires online. Invited participants needed to come in person to the study center.

### Participants’ profiles

The characteristics of the participants to the random sample (N = 638) and selenium sub-study (N = 109) are presented in Table 5, and are compared with the corresponding available characteristics of the target population (based on the data from the Federal Statistical Office for 2020) for the random sample. These participants all completed the study visit and therefore have laboratory results and available biobanked blood and urine samples. Women were over-represented in the random and selenium sub-study samples. The mean age and the age distribution of the participants in the random sample were similar to the ones of the general population aged 20 to 69 years. Participants to the selenium sub-study were substantially younger than the ones in the random sample.

**Table 5:** Characteristics of participants from the random sample and selenium sub-study with study visit compared to the general population of the cantons VD and BE.

	Random sample (N = 638)	Selenium sub-study (N = 109)	General population VD + BE (N = 1’201’921*)
**Gender**			
Men	297 (46.6%)	30 (27.5%)	597’525 (49.7%)
Women	341 (53.4%)	79 (72.5%)	604’396 (50.3%)
**Age (years)**			
Mean (SD)	46.21 (13.49)	33.65 (10.73)	44.35 (13.76)
**Age categories (years)**			
20-29y	93 (14.6%)	48 (44.0%)	219’420 (18.3%)
30-39y	124 (19.4%)	36 (33.0%)	256’915 (21.4%)
40-49y	126 (19.7%)	14 (12.8%)	251’607 (20.9%)
50-59y	171 (26.8%)	6 (5.5%)	268’671 (22.4%)
60-69y	124 (19.4%)	5 (4.6%)	205’308 (17.1%)
**Language**			
French	230 (36.1%)	76 (69.7%)	
German	253 (39.7%)	3 (2.8%)	
Italian	17 (2.7%)	7 (6.4%)	
Other	68 (10.7%)	21 (19.3%)	
Swiss-German	70 (11.0%)	2 (1.8%)	
**Nationality**			
Swiss	463 (72.6%)	61 (56.0%)	
Swiss and one other	113 (17.7%)	26 (23.9%)	
Other nationality	62 (9.7%)	22 (20.2%)	
**Net monthly household revenue**			
< CHF 3’000	20 (3.1%)	22 (20.2%)	
between CHF 3’000 and 4’500	52 (8.2%)	12 (11.0%)	
between CHF 4’500 and 6’000	99 (15.5%)	13 (11.9%)	
between CHF 6’000 and 9’000	148 (23.2%)	26 (23.9%)	
between CHF 9’000 and 11’000	90 (14.1%)	13 (11.9%)	
> CHF 11’000	157 (24.6%)	15 (13.8%)	
Prefer not to answer	72 (11.3%)	8 (7.3%)	
**Highest level of education**			**
Primary school	17 (2.7%)	0 (0.0%)	1.3%
Secondary school	21(3.3%)	1 (0.9%)	11.5%
Highschool	23 (3.6%)	7 (6.4%)	5.0%
Apprenticeship	221 (34.6%)	12 (11.0%)	36.3%
Bachelor/Diploma	135 (21.2%)	33 (30.3%)	26.7%
Master/License	151 (23.7%)	48 (44.0%)	11.6%
Doctorate/PhD	27 (4.2%)	7 (6.4%)	2.7%
Other	35 (5.5%)	1 (0.9%)	5.0%
Prefer not to answer	8 (1.3%)	0 (0.0%)	

* Data from the Population and Households Statistics of the Federal Statistical Office: permanent resident population 20–69 in the cantons of Vaud and Berne, all nationalities with B or C permits.

**Data from the structural survey of the Federal Statistical Office: permanent resident population 20–69 in Switzerland in private households, all nationalities with B or C permits.

N-miss = 0. No missing data for any of the characteristics and sample type.

The characteristics of participants from the random sample -0 and the self-selected sample are listed in S3 Table. Women were over-represented in the self-selected sample and self-selected participants were slightly younger than participants from the random sample.

### Characteristics of non-participants

Across the two study centers, 42 non-respondent (NR) questionnaires were completed by phone interview. Out of the 42 persons responding to the NR-questionnaires, 59.5% were women, the mean age was 56.8 years, 21% reported regularly smoking, and the most frequent highest education degree was apprenticeship (35.7%). The most important reasons for refusing to take part in the study were the lack of time (33.3%) and the lack of willingness to come to a study center for a medical visit (31.0%). In 26.2% of the cases, other unspecified reasons were given.

### General health and willingness to participate to a national cohort

**Fig 3:**
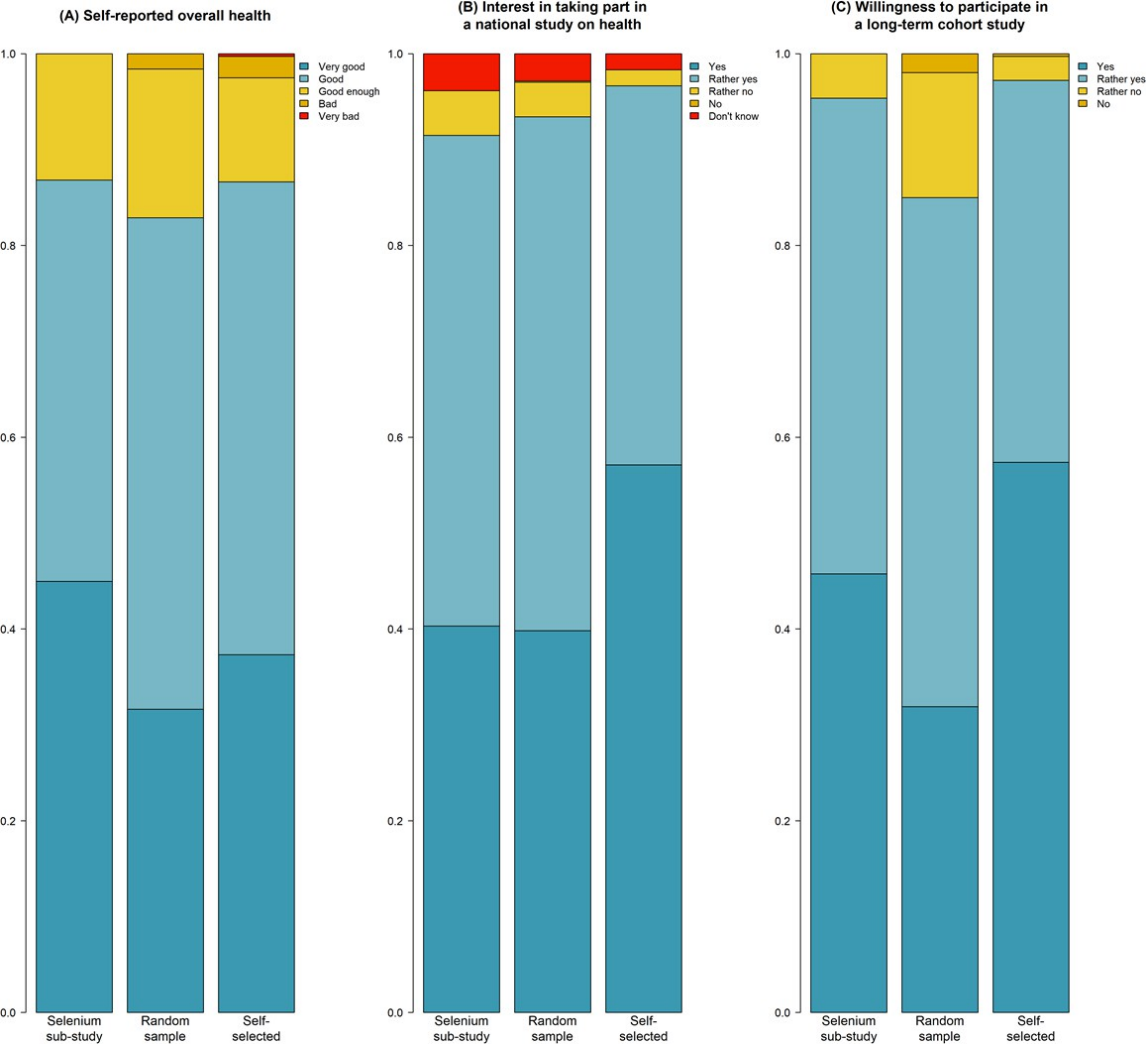
General health and intention to take part to a national study on health or a long-term cohort study, stratified by recruitment channel.

Self-reported general health status was predominantly “very good” or “good” in all three samples (86.8% in the selenium sub-study, 82.9% in the random sample and 86.6% in the self-selected sample (Fig 3(A)). The interest in taking part in a national study on health was high to very high for 91.5% in the selenium sub-study, 93.4% in the random sample and 96.7% in the self-selected sample (Fig 3(B)). The willingness to participate in a long-term cohort study was high to very high in 95.4% of participants in the selenium sub-study, 85.0% in the random sample and 97.2% in the self-selected sample (Fig 3(C)). The exact counts and percentages for each category are listed in S4 Table. The willingness of the participants to undergo the proposed actions, i.e. answering questionnaires, going to a center for a clinical evaluation, and providing biological samples is evaluated in another work using qualitative methods (Buehler et al., 2022, submitted).

### Motivations to participate to a long-term national study on health

**Fig 4:**
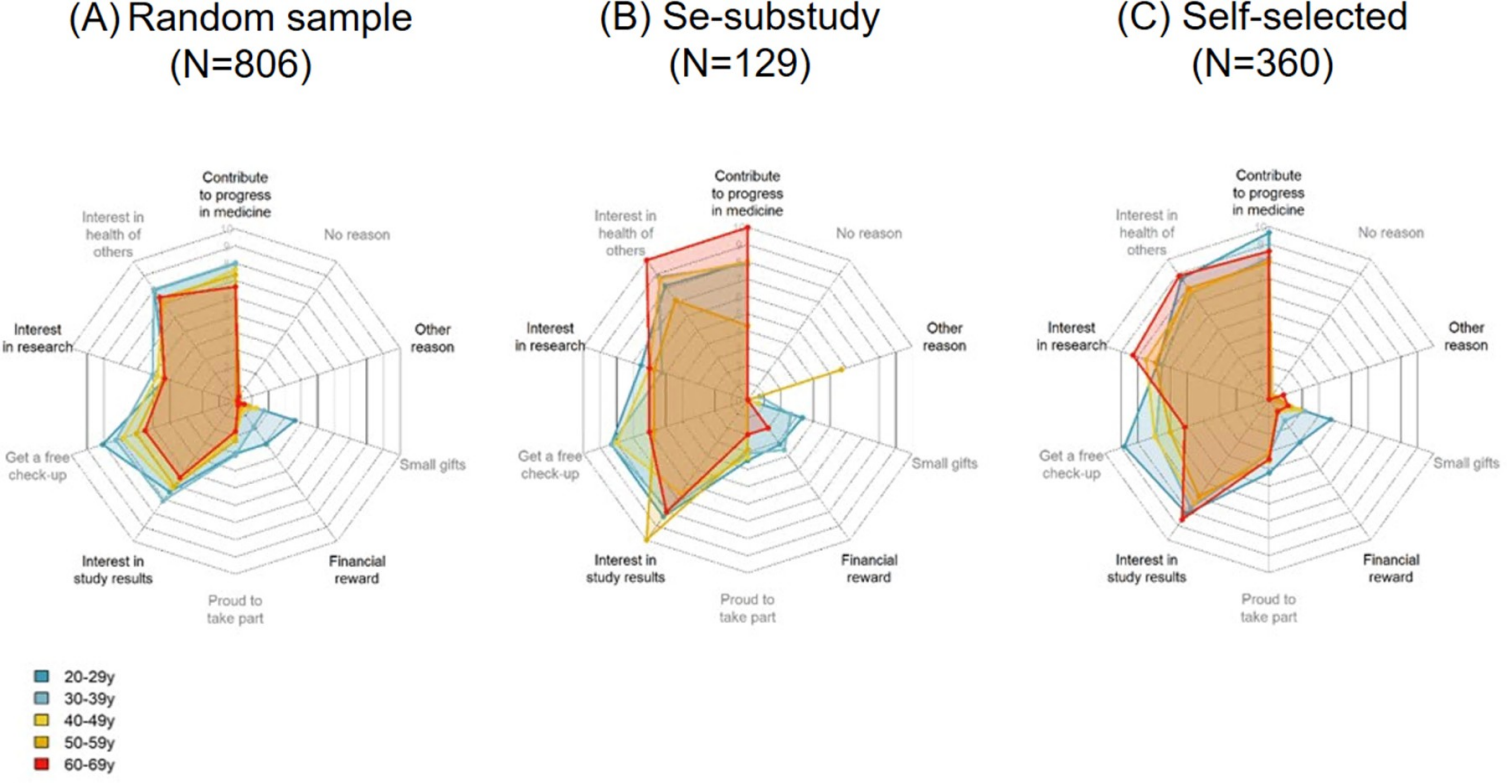
Radar plots of the reasons to take part in a long-term national cohort, by recruitment channel and age groups. Radar plot displaying the degree of agreement with proposed motives for taking part in a long-term national cohort study based on questionnaires’ responses from all participants of the random, selenium sub-study and self-selected samples, according to the age category. Options were given to participants to answer yes or no for each motive. The percentage of yes is reported here, each line corresponding to a 10% percent increase (0% in the center, 100% in the outer circumference).

The online self-administered questionnaires explored the general acceptance of public health research and more specifically the willingness to take part in a long-term national study using 10 statements, which can inform on the expected participation in a large-scale population-based cohort in Switzerland, and on opportunities for increasing the participation rate. In the random, selenium sub-study and self-selected samples (Fig 4 and S5 Table), the most frequently reported reasons to take part in a long-term national study on health were the wish to contribute to the progress of medicine (75.2%, 78.3%, 83.9%, respectively), to improve the health of others (75.7%, 84.5%, 80.0%, respectively), followed by the opportunity to get a free medical check-up (66.6%, 80.6%, 68.9%, respectively) and interest in the study results (62.5%, 80.6%, 76.4%, respectively). By contrast, financial reward (11.4%, 27.1%, 13.9%, respectively) and small gifts (14.5%, 24.8%, 20.8%, respectively) were less frequently mentioned as reasons to be willing to participate.

The interest in getting a free medical check-up was strongly associated with age and was much higher among young participants than among older ones (80.7% among the 20–29 years to 55.0% among the 60–69 years, P for trend < 0.001 in the random sample with similar results for the 2 other samples) (S5 Table). Similarly, financial reward and small gifts were more frequently listed as motivations to participate by young people than by older ones (P for trend < 0.001 in all 3 samples).

### Reasons for refusing to participate to a long-term national study on health

Reasons for refusing to participate to a long-term national study on health are reported in S1 Fig. with counts and percentages presented in S6 Table. In the random, selenium sub-study and self-selected samples, the main reasons for refusal were fear of data misuse (32.1%, 55.8%, and 38.6%, respectively), fear of misuse by the pharmaceutical industry (28.4%, 59.7%, and 34.2%, respectively), lack of time (33.6%, 34.9%, and 31.7%, respectively) and concern over data protection (24.8%, 38.8%, and 31.7%, respectively).

## Discussion

This pilot study initiated by the FOPH, in collaboration with academic partner institutions, aimed to set the ground for a large-scale long-term national population-based cohort, setting up key infrastructure components of study centers, standard operating procedures, biosample storage in a centralized biobank and quality assurance in the multilingual and multicultural Swiss environment.

The governance of the project turned out to be a key component of a successful implementation of data collection using standardized procedures in two different study centers, one in the French-speaking region (city of Lausanne, canton of Vaud) and the other in the German-speaking region (City of Berne, canton of Berne). Extrapolating from the pilot phase to a national project with even more players and interests, it will be crucial to establish clear responsibilities and decision pathways.

Large-scale population-based cohorts run in other countries have set up different governance structures. The UK Biobank is governed by the UK Biobank Board, composed of members from academia, charitable organizations and the industry, supported by multiple committees and expert advisory boards, and involves extensive consultation with the public and scientific [15]. The German National Cohort (NaKo) is a joint interdisciplinary network of scientists from the Helmholtz and the Leibniz Association, universities, and other research institutes [30, 31]. The French CONSTANCES population-based cohort was founded in its pilot phase by the “Direction générale de la santé” of the Ministry of Health with the support of different ministries, national institutes as well as the National Health Insurance Fund [32]. The innovative US “All of Us” research program led by the National Institutes of Health is a cloud-based platform connecting multiple US-based academic non-for-profit institutions and focusing on precision medicine [33]. The China Kadoorie Biobank (CKB) [34] is governed by a collaborative group, including an international steering committee and local coordinating centers. The International Hundred Thousand Plus Cohort Consortium (IHCC) [35] formed in 2020 includes 103 cohort from 43 countries with data on 50 million participants worldwide and has established policies for data sharing, findability and interoperability as well as collaborative publications, while keeping high independence at single cohort level. The governance model for Switzerland will need to take into account the specific federal and decentralized structure as well as the multicultural and multilingual context of the country. A clear definition of roles and responsibilities needs to be set up by public funders, including models for public-private partnerships and funding, as put in place within the Corona Immunitas project [36].

The participation rate of 14.0% for the random population-based sample is not optimal in view of creating a representative sample of the population aged 20 to 69 years, including socio-economic background. However, the participation rate is higher than those in other large population studies in neighboring countries: e.g., the participation rates of the UK Biobank was 5.5% [37], and it was 7.3% in the CONSTANCES study in France [38]. The NaKo study reached a participation rate of 17% at baseline [30], which is comparable to what was achieved in this pilot study with a consequent follow-up (e.g. phone calls) of the prospects as observed in the early recruitment phase. Several explanatory factors could play a role in the participation, with factors related to the study design itself (length of the questionnaires and of the study visit, types of exams), related to the recruitment strategy (sample characteristics, incl. availability of a phone number, follow-up) or to the study organization and “marketing” (accessibility of the study centers, interfaces, communication channels). The proportion of participants lost after registration could point towards too long questionnaires, poorly worded or disturbing questions or an interface to be optimized. The participation rate fluctuated throughout the sending waves, indicating seasonal and circumstantial variations. With a forced break between March and October 2020, the recruitment of the pilot phase was affected by the sanitary situation, the study teams being partly reassigned to priority tasks and some participants being reluctant to present themselves at a study center. The results indicate that with the availability of the phone number from the random sample and with a consequent and systematic follow-up of the prospects, the participation rate could be increased to up to 20%. The observed differences between the regions, the age categories and the income categories indicate that participation might also be further increased with differentiated communication strategies. The fluctuations between the sending waves also show that more efforts, respectively resources should be assigned for the follow-up of the study prospects in order to reach a representative sample and to build a reliable non-respondent model. The drop-out rate suggests also that some adaptations of the study tools might increase the participant-friendliness of the study and thus the participation rate. Options to improve participation and to ensure a well-balanced sample in terms of socio-economic status (SES) might consist in the adaptation of the study tools (such as extended schedules, broader use of apps) and with different levels of implications in the study in a modular approach (such as giving the option to fill questionnaires only, or to provide biological samples from home). The importance of incentives such as getting a full medical consultation and receiving the study results or direct financial incentives will be explored within non-respondents and participants of different categories (e.g. SES, age) with qualitative research.

The achieved participation rate in this pilot study underscores the importance of running complementary recruitment channels in parallel in order to more efficiently target specific sub-populations, such as those living in situations of vulnerability, to answer questions of public health interest and relevance. In this pilot study, we explored the feasibility of running different parallel sub-studies, which turned out to be not only possible, highly feasible, but also cost-efficient because tools can be shared and/or reused: the same data management tools can be used and the collected biosamples can serve additional purposes. Furthermore, such sub-studies generate data that are highly interoperable with the main population-based sample, hence allowing for valid comparisons with a population-based control group and better use of the publicly funded collected research data. Convenience samples are easier and less costly to recruit, but this comes at the price of uncertain external validity. The availability of the population-based component however allows assessing to what extent any given convenience sample is not representative. If the foreseen national cohort includes a sample size large enough, complemented by multiple targeted sub-studies, the value of the collected data will be very high even in the presence of selected participation biases.

Compared to the general population aged 20 to 69 years, the random population-based sample was biased towards elderly people, women as well as highly educated people. This is not surprising and commonly encountered in such population-based studies. Solutions to such participation biases are to calculate weights from the representative random population sample and run weighted analyses correcting for the observed biases. In the presence of low participation rates, such weighted analyses however cannot adequately compensate for the encountered biases.

Randomly selected participants differed from self-selected participants, by reporting less interest in taking part in the long-term study and by their inner motivations to participate that tended more towards the greater goods than their specific situation. In addition, the self-selected sample was clearly biased towards women, and was also slightly younger than the random sample—the latter supposedly less familiar with internet-based approaches—and of higher SES. These results confirm the importance of using a randomly selected sample, in particular to ensure that all SES strata are well represented; even though it is more challenging to achieve a high participation rate.

The main reasons evoked by the study participants not to take part in a long-term national cohort were the effort associated with visiting a study center and more generally the lack of time for taking part, potential data protection issues and the fear that data would be misused. Of these three reasons, in the non-respondent questionnaire only the lack of time was given as the reason for not taking part. The answers of persons who actually participated to the study, with thus a mainly positive attitude towards a health study do not reflect properly the effective reasons not to take part. To draw sound conclusions on this question, other approaches are needed, with more efforts to be placed on non-respondents.

A very large proportion of participants to the random, selenium sub-study and self-selected samples reported being in good to very good health. The result from the random population-based sample is similar to the one obtained in the Swiss Health Survey conducted in 2017 with 84% of women and 86% of men reporting to be in good or very good general health [39]. The self-reported general health was higher in the self-selected and selenium sub-study samples, which likely reflects a healthy participation bias.

In conclusion, the Swiss health study pilot phase shows great promise towards the success of a long-term national population-based cohort. Widely accessible high-quality data of public health relevance was generated. There is high interest of the general population in taking part to a national cohort on health. The global acceptance of the study and its procedures was very high. Challenges reside in setting up an efficient governance model, achieving a higher participation rate and external validity.

## Supporting information

S1 Fig Radar plot displaying the degree of agreement with proposed motives for not wanting to take part in a long-term national cohort study, based on questionnaires’ responses from all participants of the random, selenium sub-study and self-selected samples, according to the age category. Options were given to participants to answer yes or no for each motive. The percentage of yes is reported here, each line corresponding to a 10% percent increase (0% in the center, 100% in the outer circumference).
(TIF)

S1 Table Participation, refusal and questionnaires’ completion rates across mailing waves in the random population sample. (TIF)

S2 Table Participation rate by phone number availability in the random population sample. (TIF)

S3 Table Characteristics of participants from all recruitment channels. * Data from the Population and Households Statistics of the Federal Statistical Office: permanent resident population 20–69 in the cantons of Vaud and Berne, all nationalities with B or C permits. **Data from the structural survey of the Federal Statistical Office: permanent resident population 20–69 in Switzerland in private households, all nationalities with B or C permits.
(PDF)

S4 Table Partitioning of study participants into four, resp. five categories of response regarding general health and intention to take part to a national study on health or a long-term cohort study, stratified by recruitment channel. (PDF)

S5 Table Motivations to take part to a long-term national cohort, by recruitment channel and by age (numerical results corresponding to Fig 4). (PDF)

S6 Table Reasons to refuse to take part in a long-term national cohort, by recruitment channel–(numerical results corresponding to S1 Fig). (PDF)

S7 Table STROBE checklist v4 combines PLOSMedicine. (DOCX)
