# Supplementary figures and images for: Towards a Swiss health study with human biomonitoring: Learnings from the pilot phase about participation and design
Source: PLoS One. 2023 Jul 31;18(7):e0289181. doi: 10.1371/journal.pone.0289181 (PMC10389725; doi:10.1371/journal.pone.0289181)

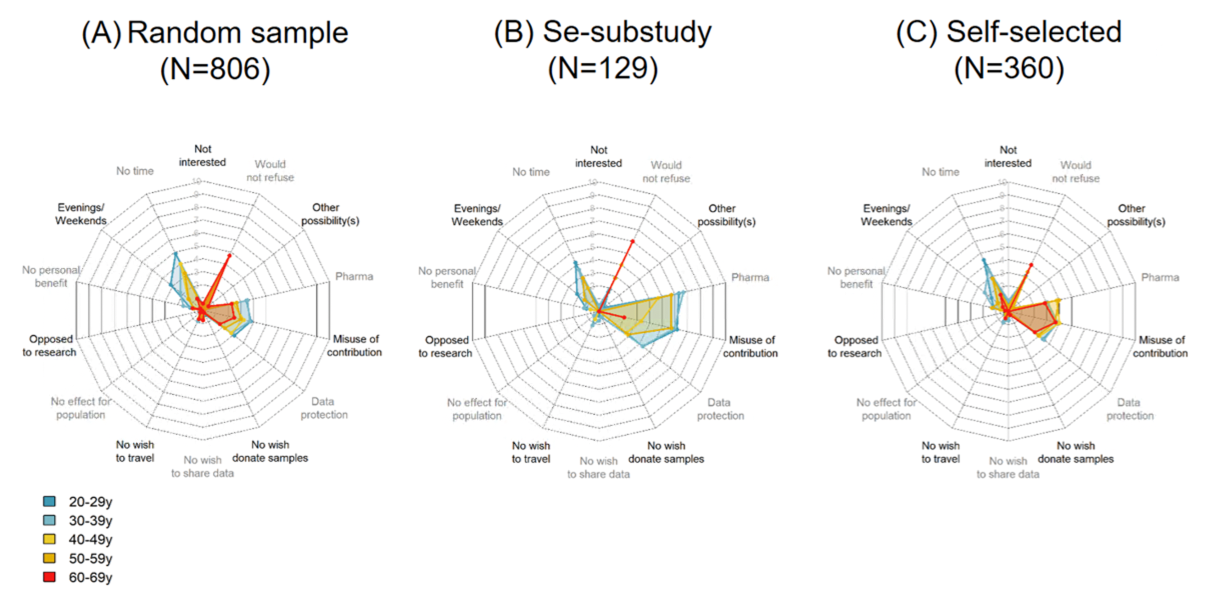

Supplement: S1 Fig — Options were given to participants to answer yes or no for each motive. The percentage of yes is reported here, each line corresponding to a 10% percent increase (0% in the center, 100% in the outer circumference). (TIF) [file pone.0289181.s001.tif]

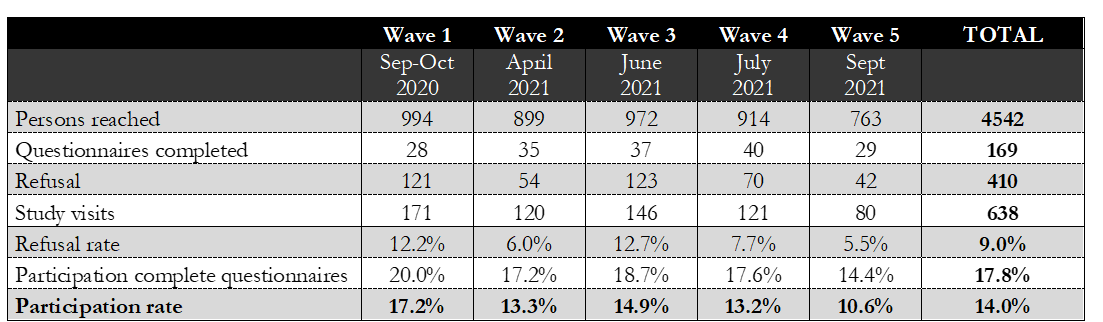

Supplement: S1 Table — (TIF) [file pone.0289181.s002.tif]

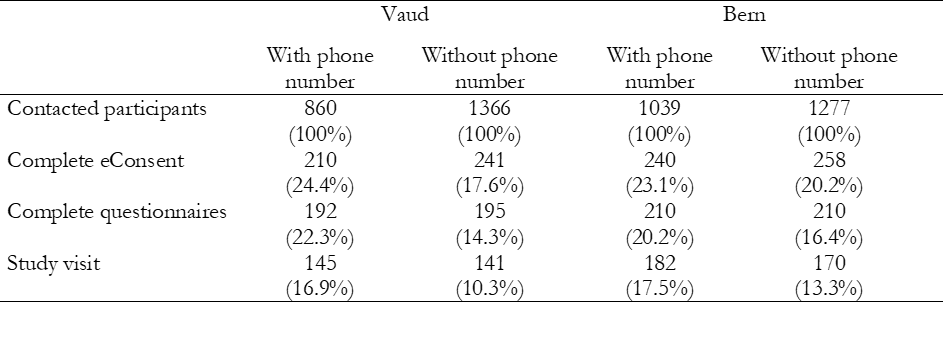

Supplement: S2 Table — (TIF) [file pone.0289181.s003.tif]
