# Supplementary material for: Towards a Swiss health study with human biomonitoring: Learnings from the pilot phase about participation and design
Source: PLoS One. 2023 Jul 31;18(7):e0289181. doi: 10.1371/journal.pone.0289181 (PMC10389725; doi:10.1371/journal.pone.0289181)
Supplement: S3 Table — * Data from the Population and Households Statistics of the Federal Statistical Office: permanent resident population 20–69 in the cantons of Vaud and Berne, all nationalities with B or C permits. **Data from the structural survey of the Federal Statistical Office: permanent resident population 20–69 in Switzerland in private households, all nationalities with B or C permits. (PDF) [file pone.0289181.s004.pdf]

| | Random sample 0 (N=69) | Random sample (N=806) | Selenium sub-study (N=129) | Self-selected sample (N=360) | General population VD + BE (N=1'201'921*) |
|---|---|---|---|---|---|
| **Gender** | | | | | |
| Men | 28 (40.6%) | 368 (45.7) | 33 (25.6%) | 131 (36.4) | 597'525 (49.7%) |
| Women | 41 (59.4%) | 438 (54.3) | 96 (74.4%) | 229 (63.6) | 604'396 (50.3%) |
| N-Miss | 0 | 0 | 0 | 0 | |
| **Age (years)** | | | | | |
| Mean (SD) | 44.77 (12.51) | 45.65 (13.44) | 33.09 (10.27) | 41.96 (11.96) | 44.35 (13.76) |
| N-Miss | 0 | 0 | 0 | 0 | |
| **Age categories (years)** | | | | | |
| 20-29y | 6 (8.7%) | 119 (14.8) | 60 (46.5%) | 59 (16.4) | 219'420 (18.3%) |
| 30-39y | 23 (33.3%) | 173 (21.5) | 42 (32.6%) | 106 (29.4) | 256'915 (21.4%) |
| 40-49y | 16 (23.2%) | 158 (19.6) | 15 (11.6%) | 92 (25.6) | 251'607 (20.9%) |
| 50-59y | 13 (18.8%) | 207 (25.7) | 7 (5.4%) | 68 (18.9) | 268'671 (22.4%) |
| 60-69y | 11 (15.9%) | 149 (18.5) | 5 (3.9%) | 35 (9.7) | 205'308 (17.1%) |
| N-Miss | 0 | 0 | 0 | 0 | |
| **Language** | | | | | |
| French | 36 (52.2%) | 239 (29.7) | 92 (71.3%) | 145 (40.3) | |
| German | 17 (24.6%) | 365 (45.3) | 4 (3.1%) | 122 (33.9) | |
| Italian | 1 (1.4 %) | 19 (2.4) | 8 (6.2%) | 12 (3.3) | |
| Other | 12 (17.4%) | 78 (9.7) | 23 (17.8%) | 46 (12.8) | |
| Swiss-German | 3 (4.3%) | 105 (13.0) | 2 (1.6%) | 35 (9.7) | |
| N-Miss | 0 | 0 | 0 | 0 | |
| **Nationality** | | | | | |
| Swiss | 35 (50.7%) | 567 (70.3) | 74 (57.4%) | 236 (65.7) | |
| Swiss and one other | 16 (23.2%) | 150 (18.6) | 31 (24.0%) | 77 (21.4) | |
| Other nationality | 18 (26.1%) | 89 (11.0) | 24 (18.6%) | 46 (12.8) | |
| N-Miss | 0 | 0 | 0 | 1 | |
| **Net monthly household revenue** | | | | | |
| < CHF 3'000 | 0 (0.0%) | 25 (3.1) | 25 (19.4%) | 18 (5.0) | |
| between CHF 3'000 and 4'500 | 6 (8.7%) | 62 (7.7) | 16 (12.4%) | 17 (4.7) | |
| between CHF 4'500 and 6'000 | 8 (11.6%) | 130 (16.1) | 14 (10.9%) | 44 (12.3) | |
| between CHF 6'000 and 9'000 | 13 (18.8%) | 192 (23.8) | 31 (24.0%) | 87 (24.2) | |
| between CHF 9'000 and 11'000 | 18 (26.1%) | 120 (14.9) | 15 (11.6%) | 66 (18.4) | |
| > CHF 11'000 | 17 (24.6%) | 191 (23.7) | 18 (14.0%) | 100 (27.9) | |
| Prefer not to answer | 7 (10.1%) | 86 (10.7) | 10 (7.8%) | 27 (7.5) | |
| N-Miss | 0 | 0 | 0 | 1 | |
| **Highest level of education** | | | | | ** |
| Primary school | 2 (2.9%) | 23 (2.9) | 0 (0.0%) | 4 (1.1) | 1.3% |
| Secondary school | 3 (4.3%) | 24 (3.0) | 2 (1.6%) | 1 (0.3) | 11.5% |
| Highschool | 2 (2.9%) | 31 (3.8) | 9 (7.0%) | 12 (3.3) | 5.0% |
| Apprenticeship | 16 (23.2%) | 273 (33.9) | 16 (12.4%) | 78 (21.7) | 36.3% |
| Bachelor/Diploma | 16 (23.2%) | 173 (21.5) | 40 (31.0%) | 81 (22.6) | 26.7% |
| Master/License | 22 (31.9%) | 198 (24.6) | 53 (41.1%) | 118 (32.9) | 11.6% |
| Doctorate/PhD | 4 (5.8%) | 35 (4.3) | 8 (6.2%) | 39 (10.9) | 2.7% |
| Other | 2 (2.9%) | 40 (5.0) | 1 (0.8%) | 25 (7.0) | 5.0% |
| Prefer not to answer | 2 (2.9%) | 9 (1.1) | 0 (0.0%) | 1 (0.3) | |
| N-Miss | 0 | 0 | 0 | 1 | |
