# Supplementary material for: Towards a Swiss health study with human biomonitoring: Learnings from the pilot phase about participation and design
Source: PLoS One. 2023 Jul 31;18(7):e0289181. doi: 10.1371/journal.pone.0289181 (PMC10389725; doi:10.1371/journal.pone.0289181)
Supplement: S4 Table — (PDF) [file pone.0289181.s005.pdf]

	Self-reported general health*	Interest in national study**	Interest in long-term cohort***
Random sample			
1	31.6%	39.8%	31.9%
2	51.2%	53.6%	53.1%
3	15.5%	3.60%	13.0%
4	1.61%	0.12%	1.98%
5	0%	2.9%	-
Selenium sub-study			
1	45.0%	40.3%	45.7%
2	41.9%	51.2%	49.6%
3	13.2%	4.65%	4.65%
4	0%	0%	0%
5	0%	3.88%	-
Self-selected sample			
1	37.3%	57.1%	57.4%
2	49.3%	39.6%	39.8%
3	10.9%	1.67%	2.5%
4	2.23%	0%	0.28%
5	0.28%	1.67%	-
