# Supplementary material for: Towards a Swiss health study with human biomonitoring: Learnings from the pilot phase about participation and design
Source: PLoS One. 2023 Jul 31;18(7):e0289181. doi: 10.1371/journal.pone.0289181 (PMC10389725; doi:10.1371/journal.pone.0289181)
Supplement: S5 Table — (PDF) [file pone.0289181.s006.pdf]

Random Sample	20-29y (N=119) N (%)	30-39y (N=173) N (%)	40-49y (N=158) N (%)	50-59y (N=207) N (%)	60-69y (N=149) N (%)	Total (N=806) N (%)	p-value*
**I wish to contribute to the progress of medicine**	95 (79.8)	139 (80.3)	121 (76.6)	152 (73.4)	99 (66.4)	606 (75.2)	**0.033**
**I want to help improve the health of others**	95 (79.8)	139 (80.3)	112 (70.9)	153 (73.9)	111 (74.5)	610 (75.7)	0.232
**I am interested in research and health**	51 (42.9)	87 (50.3)	75 (47.5)	91 (44.0)	64 (43.0)	368 (45.7)	0.600
**I get a free medical check-up**	96 (80.7)	126 (72.8)	108 (68.4)	125 (60.4)	82 (55.0)	537 (66.6)	**< 0.001**
**I am interested in the results of the study**	77 (64.7)	123 (71.1)	98 (62.0)	125 (60.4)	81 (54.4)	504 (62.5)	**0.035**
**I am proud to participate in an important study in Switzerland**	36 (30.3)	54 (31.2)	31 (19.6)	47 (22.7)	26 (17.4)	194 (24.1)	**0.012**
**I am motivated by a financial reward**	36 (30.3)	31 (17.9)	11 (7.0)	10 (4.8)	4 (2.7)	92 (11.4)	**< 0.001**
**I am motivated by small gifts (e.g. travel vouchers)**	43 (36.1)	30 (17.3)	20 (12.7)	16 (7.7)	8 (5.4)	117 (14.5)	**< 0.001**
**Other possibilities**	0 (0.0)	2 (1.2)	0 (0.0)	4 (1.9)	2 (1.3)	8 (1.0)	0.302
**No reason would motivate me**	1 (0.8)	3 (1.7)	7 (4.4)	3 (1.4)	5 (3.4)	19 (2.4)	0.214

**Self-selected sample**	**20-29y (N=59)**	**30-39y (N=106)**	**40-49y (N=92)**	**50-59y (N=68)**	**60-69y (N=35)**	**Total (N=360)**	**p-value**
	**N (%)**	**N (%)**	**N (%)**	**N (%)**	**N (%)**	**N (%)**	
**I wish to contribute to the progress of medicine**	57 (96.6)	87 (82.1)	74 (80.4)	54 (79.4)	30 (85.7%)	302 (83.9)	0.055
**I want to help improve the health of others**	51 (86.4)	81 (76.4)	71 (77.2)	54 (79.4)	31 (88.6)	288 (80.0)	0.350
**I am interested in research and health**	40 (67.8)	69 (65.1)	69 (75.0)	47 (69.1)	29 (82.9)	254 (70.6)	0.266
**I get a free medical check-up**	52 (88.1)	73 (68.9)	64 (69.6)	41 (60.3)	18 (51.4)	248 (68.9)	**0.001**
**I am interested in the results of the study**	48 (81.4)	82 (77.4)	68 (73.9)	47 (69.1)	30 (85.7)	275 (76.4)	0.305
**I am proud to participate in an important study in Switzerland**	25 (42.4)	33 (31.1)	28 (30.4)	24 (35.3)	12 (34.3)	122 (33.9)	0.594
**I am motivated by a financial reward**	18 (30.5)	16 (15.1)	8 (8.7)	5 (7.4)	3 (8.6)	50 (13.9)	**< 0.001**
**I am motivated by small gifts (e.g. travel vouchers)**	22 (37.3)	23 (21.7)	17 (18.5)	9 (13.2)	4 (11.4)	75 (20.8)	**0.006**
**Other possibilities**	0 (0.0)	2 (1.9)	1 (1.1)	3 (4.4)	3 (8.6)	9 (2.5)	0.068
**No reason would motivate me**	0 (0.0)	1 (0.9)	0 (0.0)	2 (2.9)	0 (0.0)	3 (0.8)	0.266

Selenium sub-study	20-29y (N=60)	30-39y (N=42)	40-49y (N=15)	50-59y (N=7)	60-69y (N=5)	Total (N=129)	p-value
	N (%)	N (%)	N (%)	N (%)	N (%)	N (%)	
**I wish to contribute to the progress of medicine**	48 (80.0)	33 (78.6)	12 (80.0)	3 (42.9)	5 (100.0%)	101 (78.3)	0.155
**I want to help improve the health of others**	49 (81.7)	37 (88.1)	13 (86.7)	5 (71.4)	5 (100.0)	109 (84.5)	0.625
**I am interested in research and health**	39 (65.0)	22 (52.4)	9 (60.0)	4 (57.1)	3 (60.0)	77 (59.7)	0.806
**I get a free medical check-up**	50 (83.3)	35 (83.3)	12 (80.0)	4 (57.1)	3 (60.0)	104 (80.6)	0.373
**I am interested in the results of the study**	50 (83.3)	33 (78.6)	10 (66.7)	7 (100.0)	4 (80.0)	104 (80.6)	0.422
**I am proud to participate in an important study in Switzerland**	21 (35.0)	13 (31.0)	5 (33.3)	2 (28.6)	1 (20.0)	42 (32.6)	0.962
**I am motivated by a financial reward**	19 (31.7)	15 (35.7)	0 (0.0)	0 (0.0)	1 (20.0)	35 (27.1)	0.031
**I am motivated by small gifts (e.g. travel vouchers)**	20 (33.3)	11 (26.2)	1 (6.7)	0 (0.0)	0 (0.0)	32 (24.8)	0.060
**Other possibilities**	0 (0.0)	3 (7.1)	0 (0.0)	4 (57.1)	0 (0.0)	7 (5.4)	**< 0.001**
**No reason would motivate me**	0 (0.0)	0 (0.0)	0 (0.0)	0 (0.0)	0 (0.0)	0 (0.0)	-
