# Supplementary material for: Towards a Swiss health study with human biomonitoring: Learnings from the pilot phase about participation and design
Source: PLoS One. 2023 Jul 31;18(7):e0289181. doi: 10.1371/journal.pone.0289181 (PMC10389725; doi:10.1371/journal.pone.0289181)
Supplement: S6 Table — (PDF) [file pone.0289181.s007.pdf]

| | Random sample (N=806) N(%) | Selenium sub-study (N=129) N(%) | Self-selected sample (N=360) N(%) |
|---|---|---|---|
| I am not interested at all | 29 (3.6%) | 3 (2.3%) | 18 (5.0%) |
| I don't have time | 271 (33.6%) | 45 (34.9%) | 114 (31.7%) |
| I would only have time to participate in the evening or on weekends | 133 (16.5%) | 19 (14.7%) | 54 (15.0%) |
| I will never personally benefit from the results | 78 (9.7%) | 12 (9.3%) | 36 (10.0%) |
| I am opposed to health research | 1 (0.1%) | 0 (0.0%) | 1 (0.3%) |
| I do not think it will improve the health of the population | 16 (2.0%) | 3 (2.3%) | 5 (1.4%) |
| I do not wish to travel to the study center | 57 (7.1%) | 7 (5.4%) | 25 (6.9%) |
| I do not wish to share my health data | 37 (4.6%) | 5 (3.9%) | 14 (3.9%) |
| I do not wish to donate blood (or other biological samples) | 13 (1.6%) | 2 (1.6%) | 7 (1.9%) |
| I am concerned that my data is not properly protected | 200 (24.8%) | 50 (38.8%) | 114 (31.7%) |
| I am afraid that my contribution will be misused (e.g. by health insurance, employer, ..) | 259 (32.1%) | 72 (55.8%) | 139 (38.6%) |
| I am afraid that my contribution will be used for the private interests of the pharmaceutical industry | 229 (28.4%) | 77 (59.7%) | 123 (34.2%) |
| Other possibility(s) | 18 (2.2%) | 4 (3.1%) | 10 (2.8%) |
| I would not refuse | 237 (29.4%) | 29 (22.5%) | 109 (30.3%) |
